# CD161^++^CD8^+^ T cells, including the MAIT cell subset, are specifically activated by IL-12+IL-18 in a TCR-independent manner

**DOI:** 10.1002/eji.201343509

**Published:** 2013-10-01

**Authors:** James E Ussher, Matthew Bilton, Emma Attwod, Jonathan Shadwell, Rachel Richardson, Catherine de Lara, Elisabeth Mettke, Ayako Kurioka, Ted H Hansen, Paul Klenerman, Christian B Willberg

**Affiliations:** 1 Peter Medawar Building for Pathogen Research, University of OxfordOxford, UK; 2 Department of Pathology and Immunology, Washington University School of MedicineSt Louis, MO, USA; 3 Oxford NIHR Biomedical Research Centre, The John Radcliffe HospitalOxford, UK

**Keywords:** CD161^++^ T cells, IL-12, IL-18, MAIT cells, T cells

## Abstract

CD161^++^CD8^+^ T cells represent a novel subset that is dominated in adult peripheral blood by mucosal-associated invariant T (MAIT) cells, as defined by the expression of a variable-α chain 7.2 (Vα7.2)-Jα33 TCR, and IL-18Rα. Stimulation with IL-18+IL-12 is known to induce IFN-γ by both NK cells and, to a more limited extent, T cells. Here, we show the CD161^++^ CD8^+^ T-cell population is the primary T-cell population triggered by this mechanism. Both CD161^++^Vα7.2^+^ and CD161^++^Vα7.2^−^ T-cell subsets responded to IL-12+IL-18 stimulation, demonstrating this response was not restricted to the MAIT cells, but to the CD161^++^ phenotype. Bacteria and TLR agonists also indirectly triggered IFN-γ expression via IL-12 and IL-18. These data show that CD161^++^ T cells are the predominant T-cell population that responds directly to IL-12+IL-18 stimulation. Furthermore, our findings broaden the potential role of MAIT cells beyond bacterial responsiveness to potentially include viral infections and other inflammatory stimuli.

## Introduction

High expression of CD161 by CD8^+^ T cells defines the overlapping Tc17 and mucosal-associated invariant T (MAIT) cell populations. Both subsets are thought to develop from a pool of precommitted CD161 high expressing T cells [1]. Human MAIT cells comprise up to ∼5% of the total T-cell population, compared with ∼0.1% invariant natural killer T (NKT) cells (Vα24-Jα18^+^), and dominate the CD161^++^CD8^+^T cell population within adult peripheral blood, representing up to 95% of this population [1,2]. Thus, these cells represent a major innate-like T lymphocyte population within the human body.

MAIT cells express a semi-invariant TCR, composed of the invariant variable-α chain 7.2 (Vα7.2)-Jα33 [1,3,4]. They are restricted to the evolutionarily conserved, nonpolymorphic MHC-related protein (MR) 1 [3–5]. Evidence from a number of groups has suggested that the source of the MR1 ligand is bacterial [6–9]. Recently, Kjer-Nielsen et al. demonstrated that the riboflavin metabolite, rRL-6-CH_2_OH, was able to stabilize MR1 and activate MAIT cells, suggesting that MAIT cells are specific for riboflavin-producing microorganisms [10].

CD161^++^CD8^+^ T cells, and the MAIT cell subset, are found at potential sites of pathogen entry within epithelia, in particular lamina propria, as well as in high numbers in the liver [4,11,12]. The MAIT cell subset has been associated with protection from bacterial infections in a number of mouse models: *Klebsiella pneumoniae* [13], *Escherichia coli* [8], *Mycobacterium abscessus* [8], and BCG [14]. Furthermore, in humans, MAIT cells have also been observed within the lungs of tuberculosis patients [15].

CD161^++^CD8^+^ T cells have also been implicated in a number of inflammatory settings independent of bacterial infection. They are enriched within the livers of patients with chronic hepatitis C (HCV) infection, autoimmune hepatitis, primary biliary cirrhosis, alcoholic liver disease, and nonalcoholic steatohepatitis [12,16]. CD161^++^CD8^+^ T cells have been found in the brains of patients with multiple sclerosis and have been suggested to have a pathogenic role [17]. Moreover, Tc17 cells are thought to play an important role orchestrating skin inflammation in murine models of psoriasis [18].

Overall, there is strong evidence that CD161^++^CD8^+^ T cells, including the MAIT cell subset, have an important role in inflammation, even in the absence of bacterial infection. However, the mechanism(s) driving the activation of these cells is not clear.

Given the high expression of the IL-18 receptor observed on the CD161^++^CD8^+^ T-cell population [12], we hypothesized that CD161^++^CD8^+^ T cells, including the MAIT cell subset, could be activated by cytokine stimulation with IL-12 and IL-18, as has been reported with NK cells [19]. Here, we demonstrate that not only could this population express IFN-γ in response to cytokine stimulation, but it was also the primary T-cell population to do so. Furthermore, we demonstrate that bacteria, including nonriboflavin metabolizing species, and Toll-like receptor (TLR) agonists could indirectly activate CD161^++^CD8^+^ T cells via this mechanism.

## Results

### IL-12+IL-18 stimulation specifically activates CD161^++^CD8^+^ T cells

**Figure 1:**
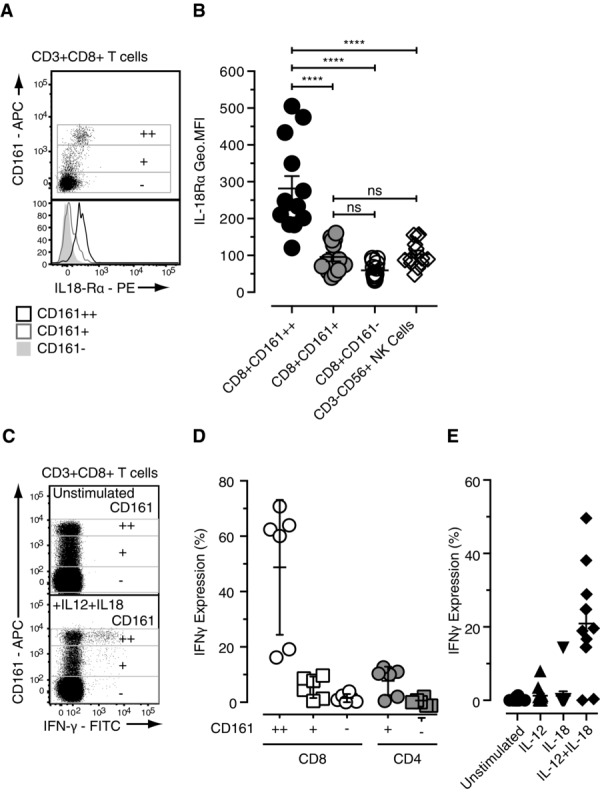
Intra- and extracellular IL-18Rα expression on CD8^+^ T-cell subsets. (A) Representative flow cyto-metry plots of IL-18Rα expression are shown. (B) The geometric MFI of IL-18Rα expression for each subset is shown (*n* = 13). (C, D) IL-12+IL-18 specifically triggers CD161^++^CD8^+^ T cells to express IFN-γ. (C) Raw flow cytometry data, as well as (D) IFN-γ expression by the different T-cell subsets after stimulation with IL-12+IL-18 are shown (*n* = 6). (E) Neither IL-12 nor IL-18 alone induces IFN-γ expression by the CD161^++^ CD8^+^ T-cell population (*n* = 10). Each symbol represents an individual sample and bars represent means and SEM. Data shown are pooled from three experiments performed. ****p < 0.0001, one-way repeated measures ANOVA with Bonferroni's multiple comparison test.

Initially, we confirmed the previous findings that CD161^++^CD8^+^ T cells expressed a significantly higher level of IL-18Rα compared with other CD8^+^T cell subsets [12]. Indeed, CD161^++^CD8^+^ T cells expressed significantly higher IL-18Rα levels compared with either CD161^+^ (*p* ≤ 0.001) or CD161^−^ subpopulations (*p* ≤ 0.001, Fig.1A and B). Interestingly, the level of expression was also almost threefold higher than on NK cells (*p* ≤ 0.001).

It is well established that the combination of IL-12 and IL-18 induces IFN-γ expression by murine NK cells and T cells [20,21]. Therefore, we asked if CD161^++^CD8^+^ T cells were more sensitive to IL-12+IL-18 stimulation compared with the other T-cell subsets. Surprisingly, only the CD161^++^CD8^+^ T-cell population responded robustly to stimulation (mean approximately 50%) (Fig.1C and D). While both CD161^+^CD8^+^ T-cell and CD161^+^CD4^+^ T-cell populations made limited responses (mean response ≤ 10%), CD161^−^CD8^+^ T-cell and CD161^−^CD4^+^ T-cell populations did not respond. Stimulation with either IL-12 or IL-18 alone did not induce IFN-γ expression from any T-cell subset (Fig.1E).

### IL-12+IL-18-induced IFN-γ expression is TCR independent

In adults, MAIT cells dominate the CD161^++^CD8^+^ T-cell population, representing approximately 87% of the total population (Fig.3A) [1]. They express a semi-invariant TCR and are restricted to MR1. However, little is known about the expression and regulation of MR1. Therefore, we investigated whether the stimulation of CD161^++^CD8^+^ T cells by IL-12+IL-18 was MR1 dependent.

**Figure 2:**
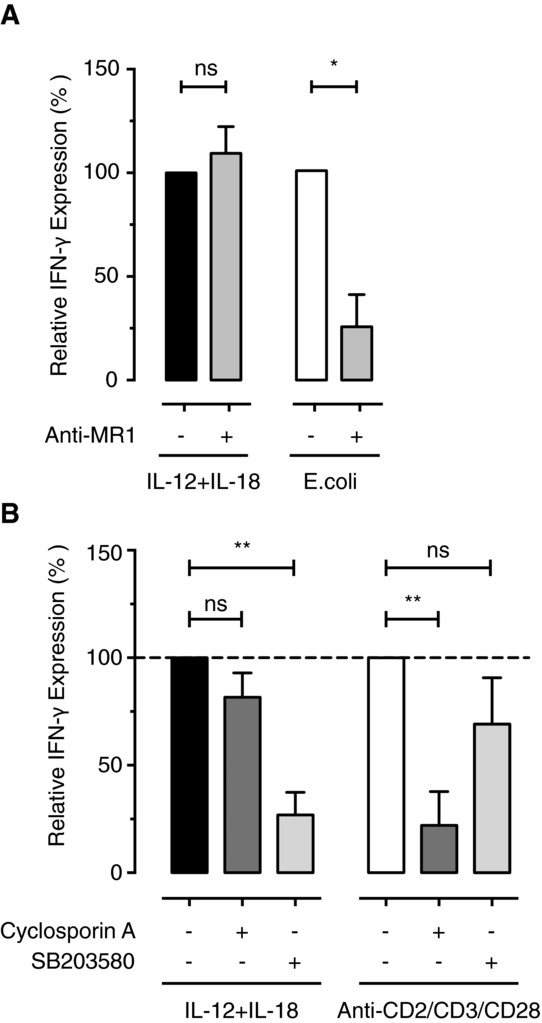
IFN-γ expression was not dependent on TCR signaling. (A) PBMCs were stimulated either with IL-12+IL-18 or with *E. coli* ± anti-MR1 (5 μg/mL) (*n* = 7). (B) PBMCs were stimulated with either IL-12+IL-18 or anti-CD2/CD3/CD28 ± inhibitors cyclosporin A or SB203580 (*n* = 7). Relative IFN-γ expression was determined by FACS. Data are shown as mean ± SEM of the indicated number of samples and are pooled from two experiments performed. *p < 0.05, **p < 0.01, one-way repeated measures ANOVA with Bonferroni's multiple comparison test.

First, PBMCs were stimulated with either IL-12+IL-18 or *E. coli* in the presence or absence of the MR1 blocking-antibody, clone 26.5 (Fig.2A) [5]. Anti-MR1 did not affect the ability of the cells to respond IL-12+IL-18, but reduced IFN-γ expression induced by *E. coli* (*p* = 0.0135).

Next, to confirm that IL-12+IL-18 stimulation was truly TCR independent, PBMCs were stimulated with either IL-12+IL-18 or anti-CD2/CD3/CD28 beads in the presence or absence of inhibitors cyclosporin A, an inhibitor of TCR signaling, and SB203580, a p38-MAPK inhibitor (Fig.2B). Cyclosporin A did not inhibit IFN-γ expression induced by IL-12+IL-18, but did significantly inhibit IFN-γ expression by anti-CD2/CD3/CD28 beads (*p* = ns and *p* = 0.001 respectively). In contrast, the p38-MAPK inhibitor (SB203580), which inhibits IL-12+IL-18 signaling [22], inhibited IL-12+IL-18-induced IFN-γ expression (*p* = 0.001), but not anti-CD2/CD3/CD28 stimulation. Thus, IL-12+IL-18-induced IFN-γ production was independent of TCR signaling.

### IL-12+IL-18-induced IFN-γ expression is not restricted to the CD161^++^CD8^+^ T cells MAIT cell subset

**Figure 3:**
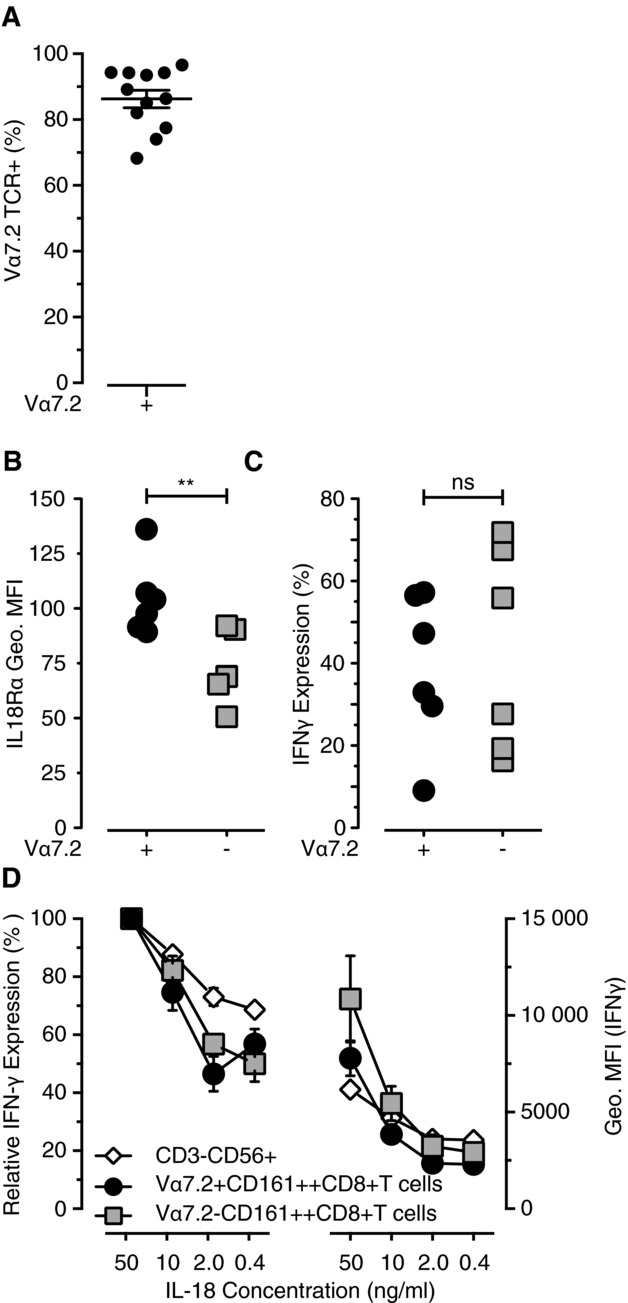
IFN-γ expression was not restricted to the MAIT cell subset of CD161^++^CD8^+^ T cells. (A) The percentage of CD161^++^CD8^+^ T cells that possess the Vα7.2 TCR. (B) The level of extracellular IL-18Rα expression was compared on the two CD161^++^CD8^+^ T cell subsets: Vα7.2^+^ and Vα7.2^−^ (*n* = 6). (C) The level of IFN-γ after IL-12+IL-18 stimulation was also measured for the two Vα7.2 subsets (*n* = 6). (D) IL-18 was titrated into PBMC cultures in the presence of IL-12. The percentage of maximum IFN-γ expression, induced by IL-18 at 50 ng/mL, was used to compare sensitivity to lower IL-18 concentrations (*n* = 6). The levels of IFN-γ expression were determined by the geometric MFI of the IFN-γ^+^ population for each cell subset (*n* = 6). (A–C) Each symbol represents an individual population. (D) Data are shown as mean ± SEM of the indicated number of samples and are from one experiment representative of at least two performed. **p < 0.01, paired *t*-test.

As IL-12+IL-18-induced IFN-γ production was independent of MR1 and TCR signaling, we questioned whether IL-12+IL-18 responsiveness was a characteristic specific to the MAIT cells, or to the CD161^++^CD8^+^ T-cell population as a whole. T cells possessing the Vα7.2 TCR dominate the CD161^++^CD8^+^ T-cell population in adult peripheral blood, ranging from approximately 70–95% in healthy individuals (Fig.3A).

We assessed IL-18Rα expression and found significantly higher levels of expression on Vα7.2^+^CD161^++^CD8^+^ T cells compared with the smaller Vα7.2^−^CD161^++^CD8^+^ T-cell subset (*p* = 0.0091, Fig.3B). However, this difference did not result in increased sensitivity to IL-12+IL-18 stimulation as both subsets responded equally to IL-12+IL-18 stimulation (Fig.3C). Furthermore, titration of IL-18 did not reveal the MAIT cell subgroup to be more sensitive to lower IL-18 concentrations compared with either the Vα7.2^−^CD161^++^CD8^+^ T-cell or NK-cell populations (Fig.3D). This was true both in terms of the percentage of cells responding and the levels of IFN-γ expressed by each cell (as determined by the geometric MFI).

Therefore, the ability to respond to IL-12+IL-18 is TCR independent. Furthermore, this is a characteristic of all CD161^++^CD8^+^ T cells, and is not restricted to just the Vα7.2^+^CD161^++^CD8^+^ T-cell (MAIT cell) subpopulation.

### Bacterial stimulation indirectly activates CD161^++^CD8^+^ T cells via IL-12 and IL-18

The key characteristic of MAIT cells is their ability to respond to bacterial infection via MR1. However, exposure of antigen presenting cells (APCs), such as monocytes, macrophages, and dendritic cells (DCs), to bacteria results in the expression of a wide range of proinflammatory cytokines, including IL-12 and IL-18 [23,24]. Thus, the total CD161^++^CD8^+^ T-cell population has the potential to respond indirectly to a wide range of microbial infections.

**Figure 4:**
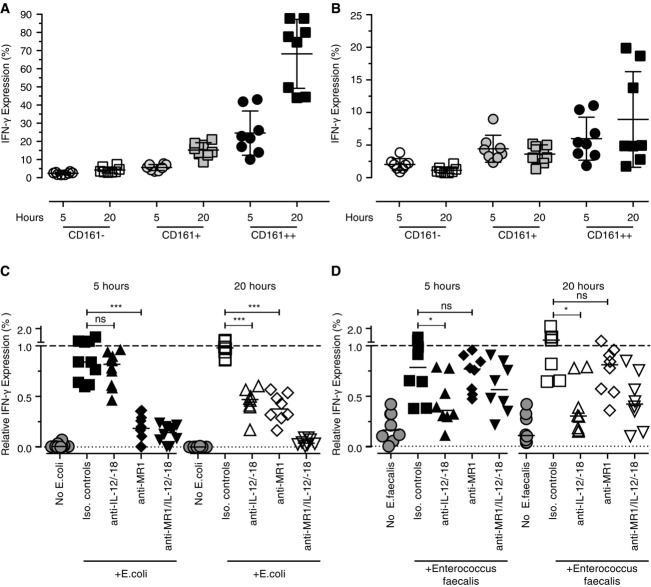
Bacterial or TLR agonist stimulation leads to IL-12+IL-18-mediated activation of CD161^++^/MAIT cells. Percentage of IFN-γ expression by CD161^++^, CD161^+^, and CD161^−^ CD8^+^ T cells cocultured with THP-1 cells exposed to (A) *Escherichia coli* or (B) *Enterococcus faecalis*, for 5 or 20 h (*n* = 8). IFN-γ expression was compared between cocultures of CD8^+^ T cells and THP-1 cells, cultured with (C) *E. coli* or (D) *E. faecalis*, in the presence or absence of blocking antibodies against IL-12, IL-18, MR1 (10 μg/mL), or isotype controls, for either 5 or 20 h. Data are presented as relative IFN-γ expression compared with that of cocultures in the absence of any antibodies. Each symbol represents an individual sample and bars represent means. *p < 0.05, **p < 0.01, ***p < 0.001, one-way repeated measures ANOVA with Bonferroni's multiple comparison test.

Using the THP-1 cell line as APCs, we first explored the contribution of MR1 and IL-12+IL-18 to the activation of CD161^++^CD8^+^ T cells by bacteria. When THP-1 cells were cultured overnight with paraformaldehyde-fixed *E. coli* and then cocultured with CD8^+^T cells for 5 h, activation was specific to CD161^++^CD8^+^ T cells and was predominantly MR1 mediated (Fig.4A and C). Blocking with anti-MR1 inhibited all IFN-γ expression, while anti-IL-12 plus anti-IL-18 blocking antibodies had no effect. However, when CD8^+^ T cells were cocultured with THP-1 cells and *E. coli* for 20 h, both MR1 and IL-12+IL-18 signaling contributed to IFN-γ expression, as blockade of either pathway reduced IFN-γ expression by ∼50%. Moreover, when THP-1 cells were exposed to paraformaldehyde-fixed *Enterococcus faecalis*, a species unable to synthesize riboflavin, only anti-IL-12+IL-18 blocked IFN-γ expression at either time point and again this was specific to the CD8^+^CD161^++^ T cells (Fig.4B and D), although the size of the response was much lower.

The differing ability of *E. coli* and *E. faecalis* treated THP1 cells to induce IFN-γ production from the CD161^++^CD8^+^ T cells was reflected in the amount of IL-12 and IL-18 secreted.

Treatment with *E. coli* resulted in considerably greater production of IL-12 compared to treatment with *E. faecalis*.

After a 20 h culture, the addition of 2 × 10^6^ and 2 × 10^7^ *E. coli* per 10^5^ THP-1 cells resulted in a mean IL-12 concentration of 2170 and 1872 pg/mL respectively. However, *E. faecalis*, at equal MOIs, only induced a mean IL-12 concentration of 81 and 253 pg/mL, respectively (Supporting Information Fig. 2A). However, no difference was observed in their ability to induce IL-18 (Supporting Information Fig. 2B).

### CD161^++^CD8^+^ T cells respond indirectly to TLR agonists

**Figure 5:**
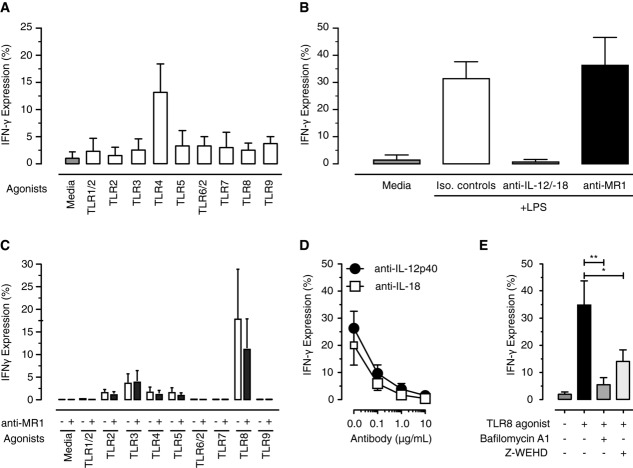
Toll-like receptor activation leads to IFN-γ expression by CD161^++^CD8^+^ T cells indirectly by IL-12+IL-18 expression. (A) THP-1 cells were stimulated overnight with TLR agonists (1–9), prior to coculture with CD8^+^ T cells. IFN-γ expression by CD161^++^CD8^+^ T cells was subsequently measured after a further 20 h incubation (*n* = 4). (B) LPS-stimulated THP-1 cells were cocultured with CD8^+^ T cells ± anti-IL-12, -IL-18, and -MR1 blocking antibodies (5 μg/mL) (*n* = 2). (C) PBMCs were stimulated for 24 h in the presence of TLR agonists (1–9) ± anti-MR1 and IFN-γ expression by CD161^++^CD8^+^ T cells measured (*n* = 8). (D) TLR8 agonist-induced IFN-γ expression following titration of blocking antibodies against IL-12p40 or IL-18 (*n* = 4). (E) TLR8 agonist signaling is dependent on TLR8 and the inflammasome. PBMCs were stimulated with the TLR8 agonist ± bafilomycin A or Z-WEHD (*n* = 7). Data are shown as mean ± SEM of the indicated number of samples and are pooled from two experiments performed. *p < 0.05, **p < 0.01, one-way repeated measures ANOVA with Bonferroni's multiple comparison test.

Bacteria are large complex microorganisms that can trigger a number of different pathogen recognition receptors, such as TLRs. TLRs are potent activators of the immune system and induce a number of proinflammatory cytokines, including IL-12 and IL-18 [25–28]. To test the importance of each TLR individually, we stimulated THP-1 cells with agonists to TLR 1–9 for 24 h, washed the cells extensively, and cocultured them with CD8^+^ T cells for 16 h. Only coculture with lipopolysaccharide (LPS) (TLR4 agonist) treated THP-1 cells stimulated IFN-γ production by CD161^++^CD8^+^ T cells (Fig.5A). This could be blocked by anti-IL-12 plus anti-IL-18 blocking-antibodies, but not by anti-MR1 (Fig.5B).

The inability of the THP-1 cells, stimulated with the other TLR agonists, to activate CD161^++^CD8^+^ T cells could reflect the TLRs expressed by this cell line. Therefore, we tested PBMCs with the different TLR agonists and assessed IFN-γ production by the CD161^++^CD8^+^T cell population. Unexpectedly, only the presence of the TLR8 agonist, ssRNA_40_, induced IFN-γ expression by the CD161^++^CD8^+^ T cells. This was not blocked by anti-MR1 (*p* ≤ 0.01, Fig.5C). However, again both IL-12 and IL-18 were required for activation (Fig.5D).

IFN-γ expression, induced by both TLR4 and TLR8 agonists, was restricted to the CD161^++^CD8^+^ T cell subset (Supporting Information Fig.3A and B). This is consistent with the previous IL-12+IL-18 stimulation data (Fig.1D).

To demonstrate that the response was TLR8 dependent, PBMCs were cultured with the TLR8 agonist plus the vacuolar-type H^+^ ATPase inhibitor, bafilomycin A1. Bafilomycin A1 significantly inhibited expression of IFN-γ by the CD161^++^CD8^+^ T cells (*p* ≤ 0.01, Fig.5E).

Furthermore, the expression of the active form of IL-18 requires caspase-1 and the inflammasome [29]. To confirm the involvement of caspase-1, PBMCs were cultured with the TLR8 agonist and the caspase inhibitor Z-WEHD. This resulted in a significant reduction of IFN-γ production by the CD161^++^CD8^+^ T cells (*p* ≤ 0.05, Fig.5E).

## Discussion

A number of studies have suggested that CD161^++^CD8^+^ T cells, and the MAIT cell subset, are associated with viral and autoimmune inflammatory conditions [12,16–18]. This has raised the question of whether these cells actively contribute to inflammation and if so by what mechanism. In this study, we have demonstrated that CD161^++^CD8^+^ T cells, including both Vα7.2^+^ MAIT cells and Vα7.2^−^ cells, are able to produce IFN-γ in an MR1-independent fashion in response to IL-12 plus IL-18. This provides a mechanism by which these cells may contribute to the pathogenesis of inflammatory disease.

IL-18 was first identified as IFN-γ inducing factor by Okamura et al. in 1995 [30]. When combined with IL-12, IL-18 has been shown to be potent inducer of IFN-γ in human NK cells and, to a more limited extent, in human T cells [31]. In this study, we have shown that the CD161^++^CD8^+^ T-cell population is the major T-cell population that produced IFN-γ in response to IL-12+IL-18 stimulation. Furthermore, the response to IL-12+IL-18 was TCR independent and was not restricted to the Vα7.2^+^ MAIT cell subpopulation, but was a characteristic of the entire CD161^++^CD8^+^ T-cell population (both Vα7.2^+^ and Vα7.2^−^). Although the MAIT cell subset expressed higher level of IL-18Rα compared to the CD161^++^CD8^+^ Vα7.2^−^ T-cell population, they did not show a greater sensitivity to IL-18 stimulation. The higher levels of IL-18Rα could be a reflection of a more activated state of the MAIT cells, as they also express higher levels of CD69 (data not shown). However, the IL-18 receptor is a heterodimer comprising IL-18Rα and IL-18β chains. Thus, the lack of an associated increase in sensitivity may be due to the levels of expression of the IL-18β chain, which has been shown to be essential for signaling [32].

IL-12 and IL-18 are proinflammatory cytokines expressed by monocytes, macrophages, and DCs in response to a range of pathogenic stimuli [25,28]. Using THP-1 cells as APCs, we demonstrated that both *E. coli* and *E. faecalis* induce sufficient IL-12 and IL-18 to activate CD161^++^CD8^+^ T cells. In the case of *E. coli*, which contains the riboflavin synthetic pathway and is able to activate MAIT cells via MR1 [8,10,33], early activation (5 h) was solely MR1 dependent, while with longer coincubation (20 h) both TCR stimulation and IL-12+IL-18 contributed to activation. In contrast, activation by *E. faecalis*, which lacks the riboflavin synthetic pathway and does not activate MAIT cells via MR1 [8,10], was solely dependent on IL-12+IL-18. The weaker stimulation seen with *E. faecalis*, compared to *E. coli*, may reflect the differing abilities of the bacteria to induce cytokine production, in particular IL-12. Of note, a recent study in mice demonstrated that MAIT cells were able to control intracellular growth of BCG in an IL-12-dependent, MR1-independent manner, suggesting that TCR-independent activation may play the dominant role in MAIT cell effector function in vivo [14]. In contrast to mice, we found that human MAIT cells require both IL-12 and IL-18 stimulation for TCR-independent activation.

TLR stimulation has also been reported to induce production of IL-12 and IL-18 [28,34,35]. Using the THP-1 model, we demonstrated that LPS, a TLR4 agonist, but not other TLR agonists, was able to stimulate IFN-γ production by the CD161^++^CD8^+^ T-cell population in an IL-12+IL-18-dependent, MR1-independent manner. This is consistent with the stronger response observed to *E. coli* than to *E. faecalis*, which lacks LPS. Alternatively, TCR stimulation may allow the CD161^++^CD8^+^ T cells to respond more vigorously to IL-12+IL-18, as suggested by the partial blockade in response at 20 h with the MR-1 blocking antibody. Furthermore, that CD161^++^CD8^+^ T cells responded to *E. faecalis* in an IL-12+IL-18-dependent manner suggests that THP1s can produce IL-12+IL-18 to stimuli other than TLR4 agonists, such as combinations of pathogen-associated molecular patterns (PAMPs). Indeed, when a TLR agonist panel was tested on PBMCs, the TLR8 agonist was the most potent indirect activator of CD161^++^CD8^+^ T cells. Again, activation was dependent upon IL-12+IL-18 production. These data differ from those presented by Le Bourhis et al., who showed that murine bone marrow (BM) derived DCs, stimulated with LPS, were unable to activate MAIT cells in an overnight coculture, although a reduction in response to *E. coli* was seen at lower multiples of infection with *MyD88*^−/−^ BMDCs [8]. This contrasts with Chua et al., who showed that BCG-mediated MAIT cell activation was dependent upon IL-12 production by infected macrophages [14]. These results are most likely due to differences in cytokines induced by different TLR agonists, or a difference in the response by the APCs involved. For example, Humann et al. demonstrated that murine BMDCs were unable to express IL-18 in response to LPS stimulation, although they were able to with *L. monocytogenes* stimulation [36]. Furthermore, it has not been reported whether murine MAIT cells express IL-18R. Therefore, differences between the data presented here and previous reports may reflect the different APCs used, as well as the ability of murine MAIT cells to respond to IL-12+IL-18. Overall, this suggests that in vivo, TCR-independent mechanisms of CD161^++^CD8^+^ T-cell activation will be dependent upon the interactions of local APCs and PAMPs.

Interestingly, in our model, IL-12+IL-18-induced IFN-γ expression by the CD161^++^CD8^+^ T cells was only seen at the 20 to 24 h time point, and not after 5 h. The lack of early IL-12+IL-18-induced IFN-γ is consistent with previous findings in NK cells and has been suggested to be due to the upregulation of transcription factors, such as IκBξ [37–39]. Furthermore, this highlights the 5 h THP1 model as a useful tool for probing the MR1-dependent activation of MAIT cells.

TCR-independent activation of CD161^++^CD8^+^ T cells provides a mechanism by which this population can respond to a wide range of pathogens, including those that do not provide a ligand for MR1. This is of particular interest for the MAIT cell populations, as the ability to respond to TLR8 agonist-induced IL-12 and IL-18 suggests that, in addition to the antibacterial role, they may play an important role in antiviral responses, as has been suggested for invariant NKT cells in in vivo infection models [40]. Interestingly, both HBV and HCV infections have been associated with IL-12 and IL-18 expression [41,42]. We have previously shown CD161^++^CD8^+^ T cells are depleted in blood of patients with chronic hepatitis C, while Tc17 cells were enriched in the liver [12]. Future studies to investigate the role of CD161^++^CD8^+^ T-cell activation by IL-12 and IL-18 in context of viral infections, such as with hepatitis C virus, would be of particular interest.

Overall, this study demonstrates that CD161^++^CD8^+^ T cells, including the MAIT cell population it encompasses, are able to respond directly to the proinflammatory cytokines IL-12 and IL-18. This implicates this cell population in a range of infectious and noninfectious inflammatory diseases. Furthermore, their ability to specifically respond to TLR8 agonist-induced cytokines, IL-12 and IL-18, suggests that in addition to their antibacterial role, they could play an important role in antiviral responses. Detailed studies on the role and activation of CD161^++^CD8^+^ T cells in both autoimmune diseases, such as multiple sclerosis [43], and infectious disease may reveal potential novel therapeutic targets.

## Material and methods

### Cell and materials

PBMCs were prepared from whole blood leukocyte cones, obtained locally from the NHS Blood and Transplant UK, by layering onto Lymphoprep (Axis Shield) gradients.

CD8^+^T cells were isolated from PBMCs by positive selection with anti-CD8 magnetic beads (Miltenyi Biotech) using an AutoMacsPro or MS columns (both Miltenyi Biotech). Purities achieved were ≥85%.

PBMCs, or isolated CD8^+^ T cells, were stimulated for 24 h with IL-12 (Miltenyi Biotech) and/or IL-18 (R&D Systems Europe) at 50 ng/mL unless otherwise stated, or anti-CD2/CD3/CD28 beads (Miltenyi Biotech). Inhibitors used are as follows: 100 ng/mL cyclosporin A (Sigma-Aldrich), 5 μM SB203580 (Sigma-Aldrich), 10 nM bafilomycin A1 (Sigma Aldrich), or 10 μM Z-WEHD (R&D Systems). TLR agonists (Source Bioscience, UK) Pam3CSK4, HKLM, Poly(I:C), LPS *E. coli* K12, Flagellin *S. typhimurium*, FSL-1, Imiquimod, ssRNA40/Lyovec, and ODN2006 were used at 1 μg/mL.

THP-1 cells (ECACC, UK) were incubated with paraformaldehyde-fixed *E. coli* (DH5α, Invitrogen) or *E. faecalis* (clinical isolate, John Radcliffe Hospital, Oxford—species identity confirmed by MALDI-ToF) at 25 bacteria per cell. Isolated CD8^+^ T cells were either added at the beginning (20 h coculture) or following overnight incubation (5 h coculture).

Blocking antibodies against IL-12p70 (R&D Systems Europe), IL-12p40/70 (eBioscience), and IL-18 (MBL International, USA) were used at 5 μg/mL, and against MR1 at 10μg/mL unless otherwise stated (clone 26.5, described [9]).

### Flow cytometry

Antibodies/dyes used were as follows: viability dye Live/Dead fixable-Near-IR (Invitrogen), CD3-eFluor450 (eBioscience), CD3-PECy7 (eBioscience), CD4-VioGreen (clone BW135/80, Miltenyi Biotech), CD8 V450 (BD), IL-18Rα-PE (Biolegend), CD161-APC (Miltenyi Biotech), IFN-γ-FITC (Miltenyi Biotech), Vα7.2-PE or -FITC (Biolegend). Gating strategy is shown in Supporting Information Fig. 1. All data were acquired on a MACSQuant (Miltenyi Biotech) and analyzed on FlowJo (Tree Star Inc.).

### Cytokine analysis

Cell culture supernatants, from THP-1 cells cultured for 20 h in the presence of either *E. coli* or *E. faecalis* at the indicated MOIs, were analyzed for total IL-12 (IL-12+p40 human ELISA kit, Invitrogen) and IL-18 (Invitrogen) ELISA detection kits, following the manufactures instructions.

### Statistical analysis

All graphs and statistical analysis were completed using Prism software version 6 (Graph Pad, San Diego, CA, USA). Statistical significance was assessed using a one-way repeated measures ANOVA with Bonferroni's multiple comparison test or a paired *t*-test.

## Abbreviations

MAIT cell: mucosal-associated invariant T cell
Vα7.2: variable-α chain 7.2
