# Supplementary material for: CD161++CD8+ T cells, including the MAIT cell subset, are specifically activated by IL-12+IL-18 in a TCR-independent manner
Source: Eur J Immunol. 2013 Oct 1;44(1):195–203. doi: 10.1002/eji.201343509 (PMC3947164; doi:10.1002/eji.201343509)
Supplement: Supplementary file 1 — Supplementary [file eji0044-0195-SD1.pdf]

European Journal of Immunology

Supporting Information

for

DOI 10.1002/eji.201343509

James E. Ussher, Matthew Bilton, Emma Attwod, Jonathan Shadwell, Rachel Richardson, Catherine de Lara, Elisabeth Mettke, Ayako Kurioka, Ted H. Hansen, Paul Klenerman and Christian B. Willberg

$CD161^{++}CD8^{+}$ T cells, including the MAIT cell subset, are specifically activated by IL-12+IL-18 in a TCR-independent manner

© 2013 The Authors. *European Journal of Immunology* published by Wiley-VCH Verlag GmbH & Co. KGaA Weinheim.

Supporting Information

Supporting Figure 1

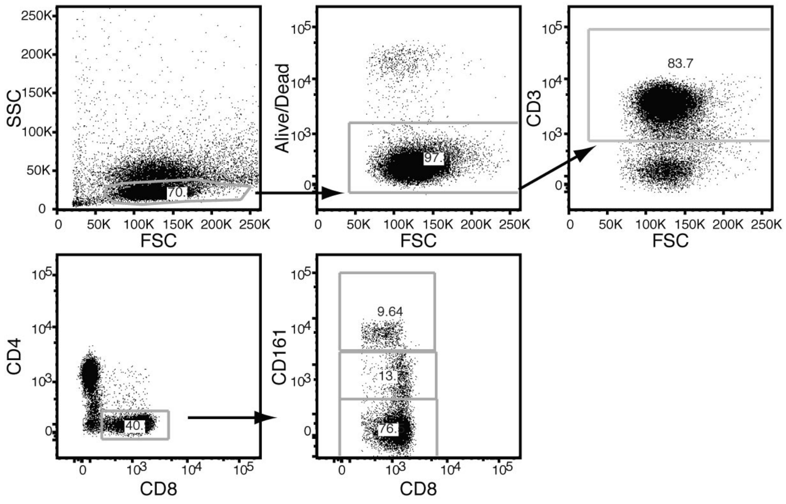

Supporting Figure 1. Flow cytometry gating strategy used to define the $CD161^{++}CD8^{+}$T-cell population.

Supporting Figure 2

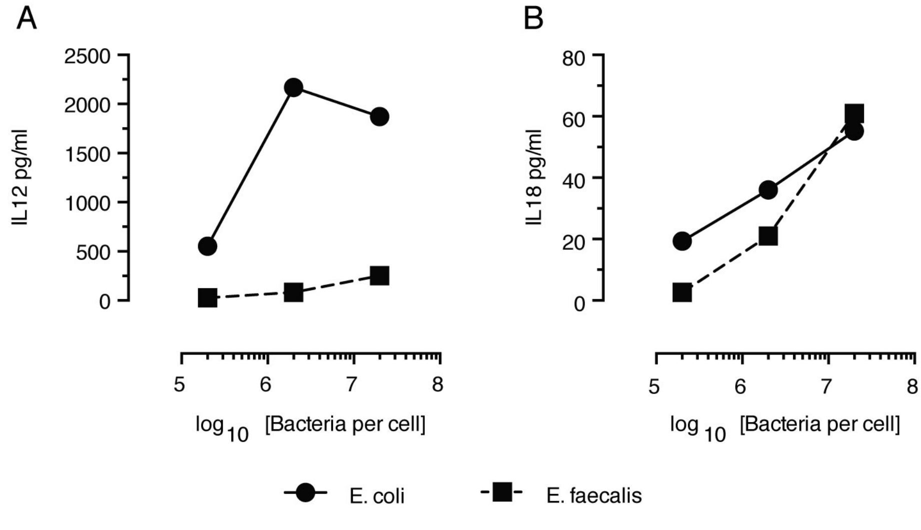

Supporting Figure 2. IL-12 and IL-18 concentrations within supernatants of THP-1 cell cultures exposed to (A) *E. coli* or (B) *Enterococcus faecalis*, at the indicated MOIs ($2x10^5$, $2x10^6$ and $2x10^7$). Cytokine concentrations were measured in the supernatants after 24-hour cultures. Data are shown as mean + SEM of duplicates and are representative plots from two independent experiments performed.

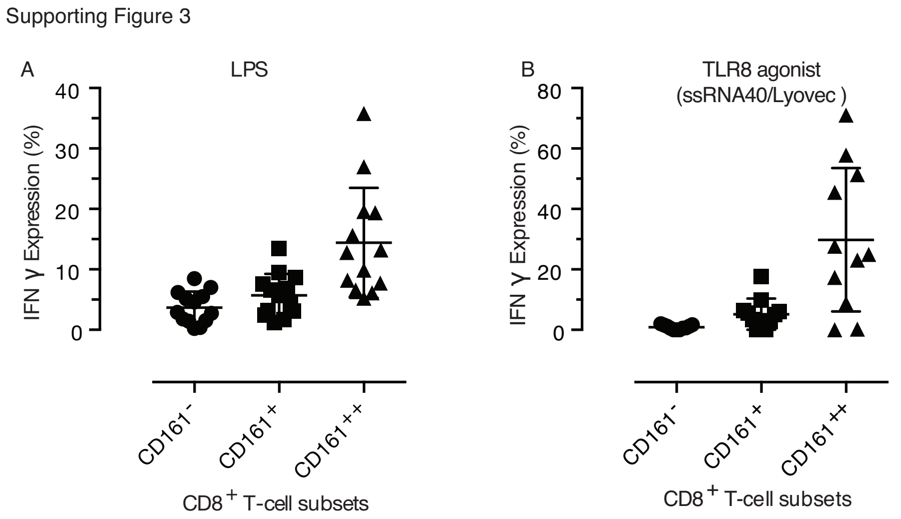

Supporting Figure 3. Toll-like receptor activation leads to IFN-γ expression primarily by $CD161^{++}CD8^{+}T$ cells, and not $CD161^{+}CD8^{+}T$ cells or $CD161^{-}CD8^{+}T$ cells. (A) THP-1 cells were stimulated overnight with TLR4 agonist (LPS), prior to co-culture with $CD8^{+}T$ cells. IFN-γ expression by the different CD161 subsets, within the enriched $CD8^{+}T$ cell population, was measured after a further 20-hour incubation (n=13). (B) PBMCs were stimulated for 24 hours in the presence of TLR8 agonist (ssRNA40). IFN-γ expression was determined from each of the different CD161 subsets within the $CD8^{+}T$ cell population (n=11). Data are shown as mean ± SEM of the indicated number of samples and are pooled from two experiments performed.
